# An Atypical Presentation of West Nile Virus With Successful Treatment After Plasma Exchange and Intravenous Immunoglobulin

**DOI:** 10.7759/cureus.24003

**Published:** 2022-04-10

**Authors:** Kevin Malone, Rahul Abraham, Grace Mccurdy, Vishal Devarkondal, Christopher M Stevens

**Affiliations:** 1 Emergency Medicine, Louisiana State University Health Sciences Center, Shreveport, USA; 2 Internal Medicine, Louisiana State University Health Sciences Center, Shreveport, USA; 3 Interventional Radiology, Louisiana State University Health Sciences Center, Shreveport, USA

**Keywords:** steroids, guillain, nile, west, adem, ivig, plasmapheresis, paralysis, neuroinvasive, west nile

## Abstract

West Nile (WN) disease is a relatively rare arboviral virus. Neuroinvasive cases of WN account for less than 1% of the total cases. The case described had difficult symptomatology and radical presentation, which included ascending paralysis. To date, there have been very few reports of West Nile cases that present with ascending paralysis. We describe the case of a 63-year-old white male who presented with a fever and proximal muscle weakness in the thighs and legs that rapidly worsened and ascended, eventually resulting in diaphragmatic paralysis. He was intubated after respiratory failure and given intravenous immunoglobulin and plasma exchange. The patient remained ventilated with persistent weakness. However, this improved after intravenous immunoglobulin and plasma exchange therapy. This case serves as a reminder to keep the diagnosis of WN on the differential, a primer on advanced treatments in the setting of aggressive atypical WN, and a lesson on similarly presenting diseases and distinguishing characteristics that may help rule out these diseases from WN.

## Introduction

The West Nile (WN) virus is an arbovirus transmitted by mosquitoes and exists within a large spatial distribution throughout the world. WN can pose significant morbidity and mortality in some cases; however, most cases present asymptomatically, with symptoms only presenting in 20 to 40% of people who are infected. Few cases present as neuroinvasive (i.e., encephalitis and meningitis); in total, around 1% of infected individuals, 10% of symptomatic individuals, and even higher in those who are immunocompromised, present with neuroinvasive WN [1]. In some cases, neuroinvasive WN can become severe enough to require critical care and may even result in death. The morbidity of patients who endure pernicious WN can be substantial, with some patients reporting physical and cognitive difficulties a year following the acute infection [1].

Typically, presentations of WN are indistinguishable from dengue fever and other viral syndromes. WN is a self-limited illness and is characterized by an abrupt onset of headache, malaise, back pain, myalgia, and anorexia. Despite the name, many patients may not display any signs of fever. Other symptoms that less typically appear are eye pains, pharyngitis, nausea, vomiting, diarrhea, and abdominal pain [1]. Herein, we describe a progressive case of neuroinvasive WN that deviated from the typical presentations described, making it hard to diagnose. Ascending paralysis, a rare finding in this situation, was seen in this patient; to date, there have been less than 20 reports demonstrating this pattern of ascending paralysis [2-9]. This case serves as a reminder to keep the diagnosis of WN on the differential, a primer on advanced treatments in the setting of aggressive atypical WN, and a lesson on similarly presenting diseases and distinguishing characteristics that may help rule out these diseases from WN.

## Case presentation

**Figure 1:**
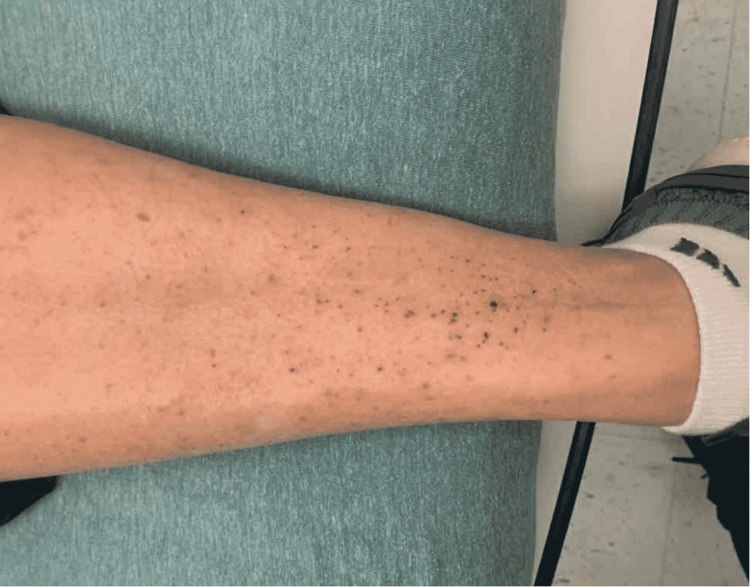
Rash seen on the patient's lower leg

A 63-year-old white male with a previous medical history significant for type 2 diabetes mellitus, diabetic neuropathy, and hyperlipidemia presented to the emergency room with three days of fever, two days of fatigue, and muscle weakness. On admission, he reported fever and two episodes of non-bilious vomiting three days prior. Additionally, he had progressive proximal muscle weakness, which he stated first appeared about three months prior and gradually got worse to the point that it limited his daily activities in the past week. He stated that he began having a difficult time getting up from bed or from the seated position in the last week, which slowly progressed to where he was unable to stand from the seated position on the day of admission. He described the weakness as a proximal weakness, mainly in the femoral region. The patient also presented with a recurrent and intermittent rash that came and went in the lower legs and feet (Figure 1, 2.).

**Figure 2:**
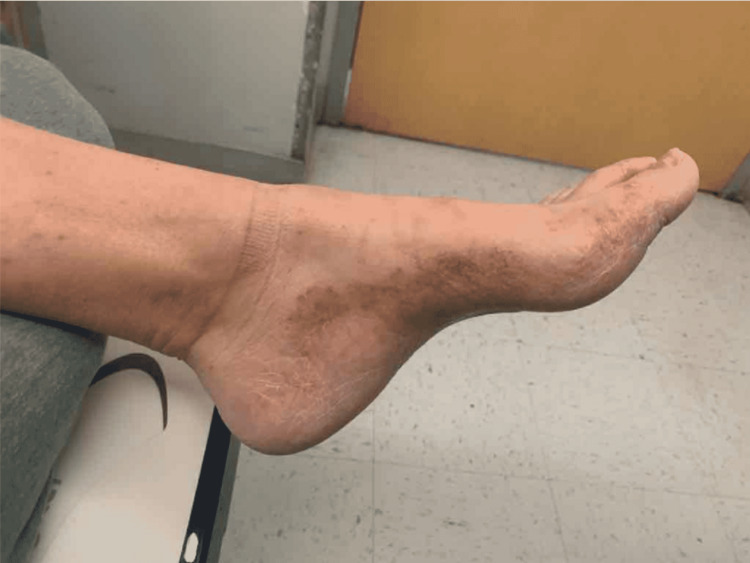
Rash seen on the patient's foot

The patient denied any diplopia, neck pain, new back pain, IV drug use, dysphagia, chest pain, and shortness of breath. There was no history of recent travel, tick exposure, and no complaints of past respiratory difficulties. Additionally, he denied all drug use and stated rare alcohol use. The patient stated he has not been sexually active since 2017, when his last long-term partner died of HIV/AIDS. Family history was significant for heart disease only.

Physical exam

Initially, the patient was febrile with a max temperature of 103 degrees Fahrenheit. Blood pressure of 103/89 mmHg, pulse of 126 bpm, respiratory rate of 20 bpm, and oxygen saturation of 98% were recorded. Rash was noted in his legs bilaterally. Cardiac and respiratory examinations were nonsignificant. No nuchal rigidity was noted, and there was no tenderness to the rash as well. Reflexes were absent in ankle jerks bilaterally, 1+knee jerks, 1+bilateral biceps, and 1+bilateral brachioradialis. The patient was oriented to person and place but not to year. The patient also had waxing and waning attention. The patient was admitted for symmetrical proximal muscle weakness, fever, nausea, and vomiting. As determined from the patient presentation and history, the causes of the patient's symptomatology were thought to be infectious versus autoimmune. The patient was officially diagnosed with West Nile disease via cerebrospinal fluid (CSF) polymerase chain reaction (PCR) and transferred to a long-term acute care facility. The patient, prior to discharge, regained baseline mental status, and muscle strength improvement was seen in the upper extremities. At the time of discharge, the patient received a percutaneous endoscopic gastrostomy (PEG) tube for eating and a tracheostomy for breathing. While total strength improved, the patient still had significant weakness in his legs and remained on tracheostomy at discharge. The chronological order of events is summarized in Table 1.

**Table 1:** Timeline of Events CPK - creatine phosphokinase, ANA - antinuclear antibodies, BiPAP - bilevel positive airway pressure, IVIG - intravenous immune globulin, CSF - cerebrospinal fluid, RBS - red blood cells, PCR - polymerase chain reaction

Day (in relation to day of admission)	Action/ event
2 months prior to presentation	Proximal muscle weakness predominately in thighs
1 week prior to presentation	Significantly daily worsening proximal weakness within thighs
3 days prior to presentation	Two episodes of vomiting and fever
Day 1	Presented to the emergency department after three days of fever, vomiting, proximal muscle weakness, and rash; Sed of 22, CPK 423, lactic 3.52; given cefepime and vancomycin
Day 2	Admitted to hospital medicine services with consults to rheumatology, neurology, and infectious disease; lactate trended down with intravenous fluids; computed tomography chest/abdomen/pelvis obtained with no significant relevant findings
Day 3	Breakthrough fever overnight of 102 degrees Fahrenheit; patient had decreased concentration, but was oriented to person, place, and time. West Nile IgM blood positive, however, can be falsely elevated. HIV negative, ANA screen negative, negative rheumatology panel. Continued antibiotics ceftriaxone, acyclovir, doxycycline, vancomycin; MRI brain without contrast showed no evidence of acute intracranial process. MRI STIR protocol, done to examine the patient's foot rash, showed increased T2 signal on both legs; suspicious of myositis.
Day 4	Patient was drowsy, and oxygen saturations were 94% overnight. Nasal cannula with 2 liters of oxygen was started. At this time, the patient notes to staff that the weakness progressed from proximal thighs to distal legs.
Day 5	In the morning, the patient had increased confusion, and tachypneic, noisy breathing on 4 liters of nasal cannula. A rapid response team was called, and the patient was started on BiPAP. Weakness continued from proximal shoulders to distal arm along with legs with increasing cognitive decline. Patient was transferred and started on IVIG for acute inflammatory demyelinating polyneuropathy after options of both plasma exchange plex and IVIG were discussed with the patient's family preferring to start IVIG.
Day 6	Increasing shortness of breath, hemodynamic instability, and cognitive decline; patient intubated due to diaphragm paralysis and placed on pressors. Attempted lumbar puncture by hospital team and ICU team was unsuccessful. Neurology interventional radiology consulted for lumbar puncture; plasma exchange with subsequent IVIG (second infusion) was given. Electromyography (EMG) study was nonsignificant.
Day 7	Plasma exchange
Day 10	Lumbar puncture completed by neurology interventional radiology
Day 11	Patient remains intubated off of pressors. CSF showed WBC 47, RBC 7650, protein 295, glucose 118. Stopped ampicillin and doxycycline; continued solumedrol and acyclovir and antibiotics; Increased levemir
Day 12	Patient remained intubated. Broad immune panel for myositis was negative. West Nile PCR positive from CSF both IgG, IgM.
Day 13	Awakens and follows commands on low dose sedation; passed spontaneous breathing trial, however negative inspiratory force was -17 cm water; all antibiotics were stopped per infectious disease's recommendations. Continued steroids
Day 14	Awake, alert, interactive, and calmer; following commands and movements in all extremities, though lower extremities slightly weaker; passed spontaneous breathing trial, but negative inspiratory force was -20; IVIG (third infusion); patient self-extubated placed on BiPAP.
Day 15	IVIG (fourth infusion)
Days 16-22	Patient showed significant improvement in work of breathing, mental status, and movement.
Day 26	Patient was discharged to long-term acute care hospital.

## Discussion

The diagnosis of West Nile may often be missed due to the inability of a proper history or the rarity of the diagnosis. This report describes a case of West Nile that was highly unique and posed several challenges in diagnosis. The patient had overlapping symptoms that are seen in other diseases (Table 2), such as a rapid progressing ascending paralysis; while this is a common feature in Guillain-Barré patients, this is a very uncommon presentation in West Nile disease and has only been reported a limited number of times [2]. The differential was initially infectious versus autoimmune causes. Initially, the proximal muscle weakness was thought to be rheumatological. Tick paralysis and West Nile disease, while on the differential, were initially low suspect. Guillain-Barré was initially suspected due to its distal extremity and ascending course. As the case evolved, the primary differential was acute inflammatory demyelinating polyneuropathy vs. chronic inflammatory demyelinating polyneuropathy. Eventually, the West Nile test resulted positive.

**Table 2:** Differentiation of overlapping symptoms seen in West Nile, Guillain-Barré syndrome, tick paralysis, and polymyositis GBS - Guillain-Barré syndrome, DTR - deep tendon reflex, CSF - cerebrospinal fluid

	West Nile	GBS	Tick paralysis	Polymyositis
Weakness	Starts on acute phase of infection	Starts weeks after acute infection, deterioration lasts days to a month usually within 2 weeks. Recovery 2-4 weeks after progression stops	Symptoms begin 2-6 days after attachment of tick	Gradual onset (insidious)
Paralysis direction		Ascending	Ascending	
Symmetry	Asymmetric, monoplegia to quadriplegia	Symmetric, proximal and distal muscles	Symmetric flaccid paralysis with loss of DTRs	Symmetrical proximal weakness
Fever	Present	No fever at onset		
CSF evaluation	Pleocytosis, elevated CSF protein.	No Pleocytosis.Elevated CSF Protein with cell count <50/mm3	Normal CSF Protein	none
Sensory	Myalgias, infrequent numbness, paresthesias, or sensory loss	Sensory loss, painful distal paresthesias	No sensory abnormalities	Generally painless (though 30% have myalgia)
Encephalopathy	Often involved	Absent	Sometimes involved	Absent

West Nile typically presents over the course of three to six days with fever, back pain, myalgia, anorexia, and headaches in about 20% of infected people, and a maculopapular rash of the face and trunk is seen in about half of patients. The rash is sometimes accompanied by complaints of dysesthesias and pruritus [10]. When present, it often appears right after a fever and, in most cases, lasts for less than one week [11]. The presence of this rash in WN patients has been associated with a decreased risk of neuroinvasive disease and death [12].

Other complications that are non-neurological include myocarditis, pancreatitis, and fulminant hepatitis, but these are rare. Less than 1% of patients with WN present with severe neurological complications. The risk of neurological complications is significantly increased with advanced age, and these patients often have increased protein and cells in the CSF [13]. Despite the name, many patients may not display any signs of fever [11,14]. Other symptoms that may less typically occur are eye pains, pharyngitis, nausea, vomiting, diarrhea, and abdominal pain [1]. Ocular manifestations that can occur include chorioretinitis and retinal hemorrhages. Uveitis, iridocyclitis, and occlusive vasculitis are among some of the other reported ocular findings in addition [15].

While not typical, meningitis and encephalitis associated with WN is a significant concern with regard to morbidity and mortality. Manifestations of meningitis related to the WN virus are similar to other viral meningitis, which include fever, headache, meningeal signs, and photophobia. The encephalitis ranges in severity, from a mild and self-limited confused state to severe encephalopathy that can lead to coma and even death, particularly in individuals over the age of fifty-five [16]. Neurological manifestations include a coarse tremor and myoclonus that are typically in the upper extremities. Parkinsonian features, such as rigidity, postural instability, and bradykinesia, may also be present [17,18].

Movement disorders, such as Parkinsonism, myoclonus, tremors, and rhabdomyolysis, have been documented in patients with WN since 2002. Some less common neurological presentations include ataxia, cranial nerve dysfunction, optic neuritis, and polyradiculopathy. Patients with hypertension or decreased immune system function may be more susceptible to central nervous system (CNS) penetration by WN. CNS involvement in WN is associated with increased mortality, long-term morbidity, and varying degrees of neurologic impairment, such as muscle weakness, mobility issues, and chronic headaches [13].

Guillain-Barré Syndrome (GBS) typically presents as a bilateral lower extremity weakness that gradually ascends to the upper extremities and face. Features common to GBS include severe respiratory muscle weakness and decreased deep tendon reflexes. Around two-thirds of patients who present with GBS follow a prior respiratory or digestive system infection. Lumbar puncture normally shows elevated proteins with normal white blood cell (WBC) count. 

Tick paralysis is easily confused with GBS. Tick paralysis is a noninfectious, neurologic syndrome that begins as acute ataxia but progresses to ascending paralysis. When recognized early, tick paralysis can result in quick recovery and can be treated with supportive measures alone. There is no fever, rash, headache, or altered mental status in the clinical presentation of tick paralysis. Tick paralysis is commonly missed because the patient does not recall any interaction with ticks, and the physician may be unable to locate the tick. Tick paralysis progresses more quickly than GBS [19].

Management

Currently, there is no established standard treatment for WN other than supportive care. Patients diagnosed with WN should be admitted to the hospital. Treatable infections and neurological conditions should be treated and/or ruled out. Patients may require intubation or mechanical ventilation if they are experiencing extreme muscle weakness. Some studies have shown some promise in using ribavirin, interferon-alpha, and pyrimidine nucleosides against WN, but more data is needed to confirm the effectiveness of these treatments [13].

In most cases, treatment of WN virus infection is primarily supportive for symptoms that may include pain control for headaches; if nausea and vomiting are present, antiemetic therapy and rehydration may be beneficial. Any patient with signs of encephalitis should be monitored for signs and symptoms of elevated intracranial pressure and seizures. For patients with poliomyelitis-like symptoms, airway protection and ventilator support may be needed.

The rationale for corticosteroid therapy in the setting of WN is to inhibit pro-inflammatory mediators that may contribute to the pathogenesis of WN in the central nervous system. Several case reports have described clinical improvement after administration of high-dose corticosteroids for a variety of WN neurological complications (e.g., acute flaccid paralysis, opsoclonus myoclonus ataxia) [20-22]. There are few studies that examine the use of steroids in patients with WN. One of the largest was a study of 228 patients with WN; of those, 65 had WN meningitis, and 53 had WN encephalitis. The findings showed no significant effect with the use of corticosteroids. In the study of patients with WN encephalitis, 17 patients received corticosteroids, and three died (18%), while nine of 48 patients who did not receive adjunctive corticosteroid treatment died (19%) [23].

Immunoglobulin is used as a treatment in an array of disorders, including primary and secondary immune deficiency and autoimmune and inflammatory disorders. There may be a theoretical benefit to administering intravenous immunoglobulin (IVIG) in patients with neuroinvasive WN who have humoral deficiencies. In the United States, high titer neutralizing antibodies have been demonstrated in plasma as a consequence of the wider prevalence of the WN virus since the original 1999 outbreak, and animal studies suggested there may be a potential role for the use of IVIG in the prevention and treatment of WN virus infections [24]. However, in a randomized, placebo-controlled, safety trial in 62 patients with neuroinvasive disease (the majority who were immunocompetent), they failed to find the benefit of a high-titer IVIG derived from donors, although this trial was designed to determine the safety and not efficacy [25].

Plasma exchange is an extracorporeal treatment that selectively removes abnormal substances in the blood that are associated with certain diseased states. It can also be used to administer new plasma or other substances and for acute disseminated encephalomyelitis (ADEM) in adults when glucocorticoids are ineffective but is are limited [26-28]. One study involving plasma exchange that included 10 patients with ADEM showed that early initiation of therapy was associated with improved outcomes [29]. There is limited data on the efficacy of plasma exchange with patients with WN, but several case reports have shown plasma exchange to lead to clinical improvement [26, 27].

## Conclusions

This case presentation was wildly unique and posed several challenges in diagnosis. This patient had a rapid progressing ascending paralysis; while a common feature in Guillain-Barré patients, this is a very uncommon presentation in West Nile disease and has only been reported in a handful of cases. Anecdotal data on treatment of West Nile shows some efficacy of intravenous immunoglobulin. Some studies have shown some promise in using ribavirin, interferon-alpha, and pyrimidine nucleosides against WN, but more data is needed to use this clinically. Corticosteroid therapy, in the setting of WN, may be used to inhibit pro-inflammatory mediators that possibly contribute to the pathogenesis of WN in the central nervous system. There is limited data on the efficacy of plasma exchange with WN patients, but several studies have shown it to be beneficial. Neuroinvasive WN has been shown to lead to Guillain-Barré in a few case reports; it is suspected that the WN infection in this report lead to Guillain-Barré, as seen in this patient. Due to the severity of the patient's presentation and the Guillain-Barré presentation, both IVIG and plasma exchange were done, which led to good outcomes in the patient.
